# Supplementary material for: Impact of Three Different Algorithms for the Screening of SSc-PAH and Comparison with the Decisions of a Multidisciplinary Team
Source: Diagnostics (Basel). 2021 Sep 22;11(10):1738. doi: 10.3390/diagnostics11101738 (PMC8534432; doi:10.3390/diagnostics11101738)
Supplement: Supplementary file 1 [file diagnostics-11-01738-s001.zip › diagnostics-1382294-supplementary.pdf]

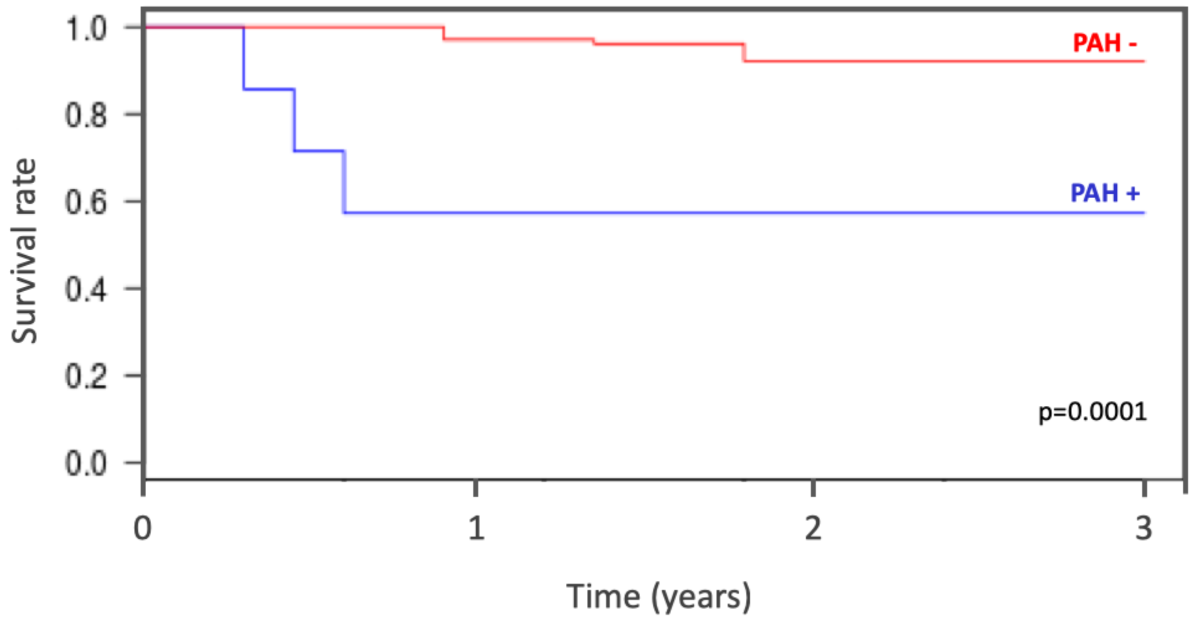

Figure S1. 3-year survival according to patients' PAH-status.

PAH-: no systemic sclerosis-associated pulmonary arterial hypertension (N=102); PAH+: systemic sclerosis-associated pulmonary arterial hypertension (N=7). P-value was calculated by the log-rank test.

Table S1. 3-year follow-up of the 9 patients without pulmonary arterial hypertension at right heart catheterization at inclusion.

Patient	TRV at baseline (m/sec)	mPAP at baseline (mmHg)	PCWP at baseline (mmHg)	PVR (WU) at baseline	Status at the end of FU	TRV at the end of FU (m/sec)	RHC controlled (yes/no)
Patient 1	3.5	18	12	1.5	dead	NA	no
Patient 2	3.2	18	9	1.5	alive	2.4	no
Patient 3	2.6	17	6	1.5	alive	2.8	no
Patient 4	3	23	9	1.3	alive	2.9	no
Patient 5	2.6	22	13	1.4	alive	2.8	no
Patient 6	3.2	35	27	1	alive	2.9	no
Patient 7	2.5	15	5	1.2	alive	2.3	no
Patient 8	3.1	21	8	2.4	alive	3.1	no
Patient 9	3.3	18	4	2.6	dead	NA	no

FU: follow-up; mPAP: mean pulmonary arterial pressure; NA: non-applicable; PCWP: pulmonary capillary wedge pressure; PVR: pulmonary vascular resistance; RHC: right heart catheterization; TRV: tricuspid regurgitation velocity; WU: wood unit.
